# Dejittering of neural responses by use of their metric properties

**DOI:** 10.1186/1471-2202-12-S1-P50

**Published:** 2011-07-18

**Authors:** Alexander G Dimitrov, Graham I Cummins

**Affiliations:** 1 Department of Mathematics and Science Programs, Washington State University Vancouver, Vancouver, WA 98686, Canada

## 

We have adapted the dejittering technique [1,2] to analyze neural response patterns. In the original manuscript [2], we developed the idea of transformation-invariant stimulus processing in order to characterize local stimulus transformations that leave the neural response invariant. Currently, the method assumes that the neural responses are identical to within a fixed temporal precision, and the stimuli that induce them are variable. In this submission, we invert the dejittering method to operate on sets of variable neural responses associated with a fixed stimulus. We now ask the question “*What are the natural transformations of neural responses that leave the neural message invariant?*” The novelty and difficulty in this adaptation is the nature of the response set, which cannot be embedded naturally in a linear vector space. For example, a naïve embedding of spike/no-spike to 0/1 in a time bin does not technically comprise a vector space, since linear combinations of such objects do not yield spike trains. Convolving spike trains with Gaussians suffers from the same constraints, while introducing additional assumptions about the metric and geometric structure of spike trains. Thus, the cost function used in stimulus dejittering (the Mahalanobis distance between a given stimulus and a Gaussian model of the set of stimuli), cannot be evaluated naturally on sets of spike trains. However, Victor and Purpura’s metric space distance (D_q_) [3], does provide an intrinsic measure of the natural cost of transformation and can replace the Mahalanobis distance in the adapted algorithm.

The metric space approach is based on providing a set of transformations that can convert one response into another. The distance between responses is the total cost of the “cheapest” set of transformations that can interconvert the signals. There have been several extension of the metric space approach, but, in the original, which we use, these transformations are the insertion or deletion of a spike (cost 1), or the translation of a spike in time (cost q per sample point of translation).

The set of D_q_ s provides a measure of the variance of a response class without the need to embed the spike trains in a space where subtraction is defined. A response class is characterized by the set of within condition distances, ⟨D_q_ (i,j)⟩j, the average distance of spike train *i* to the rest of the responses to the same stimulus. With this measure of variance, we investigate the effects of temporal shifts of individual spike trains on the overall variance of the response class. As in [2], we then minimize the joint variance ⟨D_q_(i,j)⟩_t_ + σ_t_^2^, where σ_t_^2^ is the variance of temporal shifts and ⟨D_q_(i,j)⟩_t_ is the average distance between spike trains from the same response class after temporal shifts have been applied to them.

Furthermore, a measurement of D_q_ corresponds to a set of modifications to a spike train, each of which occurs at a specific time. Thus, we can pinpoint specific regions of neural responses that are responsible for any observed large-scale temporal changes.
